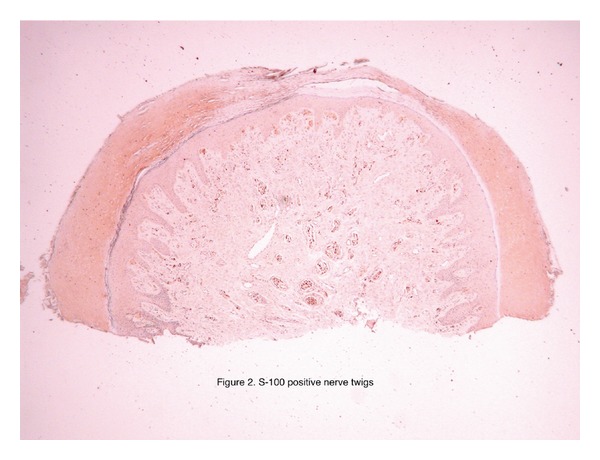# Acral Cutaneous Neural Angiomatous Hamartoma: A Variant of Palmar Cutaneous Hamartoma?

**DOI:** 10.1155/2012/945136

**Published:** 2012-10-14

**Authors:** Nebojsa Arsenovic, Anuradha Sheth

**Affiliations:** Skin Pathology, Department of Cellular Pathology, PathLinks Pathology Service, Lincoln County Hospital, Lincoln LN2 5QY, UK

## Abstract

Hamartomas are benign malformations composed of tissue elements normally found at that site, but which are growing in a disorganized fashion. Cutaneous hamartomas containing only neural and/or neurovascular elements are very rare, with very few case reports in the literature. Herein we describe a case of congenital neural angiomatous hamartoma of the skin.

## 1. Introduction

Cutaneous hamartomas (CH) are benign proliferations of normal skin elements growing in a disorganized fashion. These include a small group of rare neural hamartomas with very few case reports in the literature. CH is usually a congenital lesion, although occasionally it can be acquired. It occurs more often in children and young adults as a solitary, fleshy lesion of the skin. However, CH may appear at any age and in rare circumstances; they may be multiple lesions associated with particular syndromes. Among many examples of CH characterized by a variety of different components, neural and neurovascular hamartomas are very rare. To the authors, knowledge herein is the first case of the acral cutaneous neural angiomatous hamartoma in a child, which reveals an increased number of fine vascular elements intimately admixed with the obvious neural component.

## 2. Case Report

A 4-year-old left-handed boy presented to our outpatient clinic with a small, solitary, bluish, and slightly elevated skin lesion on the tip of his left index finger. The lesion was present since birth and did not enlarge with age. His parents had never sought any medical attention for their son's skin lesion. However, recently the boy was complaining of pain while playing and holding things in his left hand. 

Clinical examination revealed a 0.3 cm, slightly elevated, bluish lesion tender on light pressure. There was no history of significant trauma. No axillary lymphadenopathy was noted. The lesion was shaved off with the clinical differential diagnosis of a blue naevus or a myxoid cyst.

**Figure 1:**
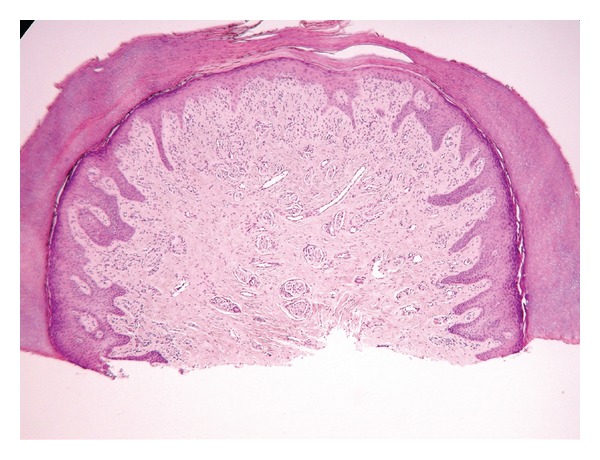


Macroscopically, the specimen consisted of a 0.3 cm fragment of skin. Microscopically, the section revealed a small fragment of acral skin. The epidermis was unremarkable. Within the dermal papillae there were regularly distributed Meissner's (tactile) corpuscles. In the papillary/reticular dermis was a poorly defined proliferation of haphazardly arranged, small nerve twigs admixed with somewhat increased number of capillary-like, thin-walled vessels some of which displayed variable size and shape (Figure 1). Some nerve twigs were in intimate contact with the vessels forming a kind of abortive neurovascular structure (Figure 1). In places, nerve twigs appeared to be protruding within the lumens of dilated vessels. There was no evidence of old or recent trauma and no fibrosis. No skin adnexae were present.

Immunoperoxidase studies were performed. The nerve twigs were positive for S100 (Figure 2). Epithelial membrane antigen decorated the perineurium around the nerve twigs. Smooth muscle actin and CD34 highlighted muscular and endothelial components of the vessels.

## 3. Discussion

Cutaneous hamartomas are rare benign lesions. They may be congenital or acquired. Various types of hamartomas have been described depending upon the components of lesion seen on the histopathological examination. Components of hamartomas usually depend on the site at which they occur. 

Neural and neurovascular hamartomas are very rare. We found very few case reports of cutaneous hamartomas containing only neural and/or neurovascular elements.

Al Habeeb et al. have described a case of cutaneous solitary neural hamartoma in a 51-year-old man [1]. This right shoulder unencapsulated lesion was composed of mature nerve bundles abnormally high within the papillary dermis, extending into the reticular dermis with a periadnexal distribution.

A case of congenital neural hamartoma on the leg of a male infanthas been described by Argenyi et al. [2]. This lesion showed an unencapsulated dermal mass composed of fascicles of spindle cells with frequent verocay body-like structures.

Another case of a cutaneous neurovascular hamartoma on the left upper back of a 29-year-old man has been described by Lee et al. [3]. Microscopically, there were irregular neurovascular structures within the deep dermis and subcutaneous fat. Bundles of spindle-shaped cells and increased numbers of small and medium-sized blood vessels were also seen.

Palmar cutaneous hamartoma was first described by Damiani and Riccioni in 1998 [4]. This lesion showed all the adnexal and mesenchymal structures which are normal constituents of the normal palmar skin. They coined a new term “neurovascular bodies” for tortuous vessels surrounded by nerve bundles seen in the lesion. They recognized that these structures were morphologically reminiscent of Sucquet-Hoyer channels which are involved in thermoregulation of the acral regions. 

Ha et al. have described two cutaneous hamartomas of the hand [5]. Neurovascular bodies were also seen in both the cases. Clinically, all three patients in these previous two case reports [4, 5] were adults who presented with painful lesions on the palmar skin. 

Our case of a painful palmar lesion in a child showed complex structures composed of bundles of nerve closely associated with thin-walled blood vessels. Occasional small blood vessels surrounded by bundles of nerve were noted. These complex structures are similar to those previously described as neurovascular bodies. No other adnexal component was present in the lesion. To the best of our knowledge, there is no previous case report of a similar congenital hamartoma with neurovascular bodies. Considering the palmar location, painful nature and presence of neurovascular bodies on microscopic examination in our case, and review of the previously mentioned case reports, this acral neural angiomatous hamartoma may be a variant of palmar cutaneous hamartoma. 

## Figures and Tables

**Figure 2:**